# Do dimensional psychopathology measures relate to creative achievement or divergent thinking?

**DOI:** 10.3389/fpsyg.2014.01029

**Published:** 2014-09-18

**Authors:** Darya L. Zabelina, David Condon, Mark Beeman

**Affiliations:** Department of Psychology, Northwestern UniversityEvanston, IL, USA

**Keywords:** creativity, psychopathology, individual differences, divergent thinking, creative achievement

## Abstract

Previous research provides disparate accounts of the putative association between creativity and psychopathology, including schizotypy, psychoticism, hypomania, bipolar disorder, ADHD, and autism spectrum disorders. To examine these association, healthy, non-clinical participants completed several psychopathology-spectrum measures, often postulated to associate with creativity: the Schizotypal Personality Questionnaire, the Psychoticism scale, the Personality Inventory for DSM-5, the Hypomanic Personality Scale, the Attention Deficit/Hyperactivity Disorder scale, the Beck Depression Inventory, and the Autism-Spectrum Quotient. The goal of Study 1 was to evaluate the factor structure of these dimensional psychopathology measures and, in particular, to evaluate the case for a strong general factor(s). None of the factor solutions between 1 and 10 factors provided a strong fit with the data based on the most commonly used metrics. The goal of Study 2 was to determine whether these psychopathology scales predict, independently, two measures of creativity: 1. a measure of participants' real-world creative achievements, and 2. divergent thinking, a laboratory measure of creative cognition. After controlling for academic achievement, psychoticism and hypomania reliably predicted real-world creative achievement and divergent thinking scored with the consensual assessment technique. None of the psychopathology-spectrum scales reliably predicted divergent thinking scored with the manual scoring method. Implications for the potential links between several putative creative processes and risk factors for psychopathology are discussed.

## Introduction

“Creativity is a divine madness, a gift from gods” Plato famously declared (cited in Neihart, 1998, p. 1), yet to this day the debate on whether creativity is associated with psychopathology remains unsettled. Research provides varying accounts of the putative associations between creativity and psychopathology, with the disparity possibly due to methodological issues, such as small, highly specialized samples of eminent creators, or dependence on subjective and anecdotal accounts (e.g., Andreasen, 1987; Jamison, 1989, 1995; Ludwig, 1995). The seemingly heterogeneous results might also be due to heterogeneous study designs and varying measurements of psychopathology and creativity (for review, Thys et al., 2014).

Nevertheless, empirical evidence exists for the association between creativity and a variety of psychopathology spectrum measures, in both clinical and non-clinical samples. Creativity, for example, is reported to relate to schizotypy and psychosis measures (e.g., Andreasen and Powers, 1975; Abraham et al., 2005). Artists are elevated on schizotypy—a set of characteristics related to schizophrenia (Eckblad and Chapman, 1983; Nelson and Rawlings, 2008). People with increased schizotypy are also better at overcoming the constraining influence of examples when trying to generate original responses on a divergent thinking task compared to people with lower schizotypy scores (Abraham and Windmann, 2008). Higher levels of psychoticism accompany a greater degree of conceptual expansion and elevated levels of originality in creative imagery (Abraham et al., 2005).

Creativity appears to also be associated with atypical attention: adults diagnosed with ADHD are suggested to have higher real-world creative achievements (White and Shaw, 2011), and outperform those without ADHD on divergent thinking tasks (White and Shaw, 2006). Creativity has also been linked with autism and the milder form of autism, Asperger's syndrome, particularly among writers, artists, and musicians, such as Lewis Carroll (Fitzgerald, 2004), Vincent Van Gogh, Glenn Gould (James, 2006), and Erik Satie (Fung, 2002).

Finally, mood disorders and creativity have been associated. Three different measures of creativity—divergent thinking, self-rated creativity, and biographical inventory of creative behaviors relate to hypomanic traits (Furnham et al., 2008). Writers are more likely to be diagnosed with unipolar depression (Kyaga et al., 2013). Bipolar disorder and schizotypy also seem to affect occupational choice and fit. Bipolar disorder, for example, is associated with engagement in creative professions in both artistic and scientific domains in a large sample (the Swedish total population register, Kyaga et al., 2013). In the same population, individuals with schizophrenia, schizoaffective disorder, unipolar depression, anxiety disorders, alcohol abuse, drug abuse, autism, ADHD, and/or suicidality had a reduced likelihood of being engaged in creative professions.

These findings may suggest that the more extreme versions of psychological disorders are not conducive to being engaged in creative professions. Milder versions, however, such as subclinical mania/depression, schizotypy, or Asperger's syndrome (possibly in combination with protective factors such as working memory, motivation, and grit (Duckworth et al., 2007), as well as other personality and environmental factors), may facilitate creative thinking without causing difficulties when engaged in the professional world. It has even been postulated that certain psychopathologies remain in the population precisely because they provide benefits of creativity to people with milder versions of the disorders, and their relatives (O'Reilly et al., 2001; Nettle, 2006). In support, psychologically healthy biological relatives of people with schizophrenia are more likely to participate in creative jobs and hobbies and tend to show higher levels of schizotypal personality traits compared to the general population (Kinney et al., 2001).

Work from multiple laboratories investigating the neuroscience of creative cognition has also suggested a link between psychopathology and creativity. Higher divergent thinkers, for instance, have lower levels of fractional anisotropy within left inferior frontal white matter (Jung et al., 2010), similar to people with schizophrenia and bipolar disorder (McIntosh et al., 2008; Sussmann et al., 2009). Additionally, both people with increased schizotypy and people with higher divergent thinking scores (emphasizing originality) exhibit reduced deactivation of the right precuneus when generating ideas (the right precuneus is thought to be responsible for gathering external and internal information Fink et al., 2014), thus both groups show similar brain patterns during idea generation.

Although numerous measures of creativity exist, divergent thinking tests and assessments of real-world creative achievements are the two commonly used measures. Divergent thinking tests assess the ability to generate many novel and appropriate responses to a given problem within a limited time (e.g., Guilford, 1967; Torrance, 1974; Goff and Torrance, 2002). A common example is the alternate uses task, which requires generating creative uses for common objects such as a brick. The process of divergent thinking corresponds to the general concept of creative idea generation. There are many possible responses to this task and people differ in the fluency (number of responses), and originality/novelty of their responses (Guilford, 1950; Runco and Acar, 2012). Divergent thinking is thought to rely on cognitive processes such as “the retrieval of existing knowledge from memory and the combination of various aspects of existing knowledge into novel ideas” (Paulus and Brown, 2007, p. 252; also see, Mednick, 1962).

Creative achievement questionnaires tally creative behaviors and outcomes. The Creative Achievement Questionnaire (CAQ; Carson et al., 2005) prompts participants to indicate prior achievements of various types in 10 (artistic and scientific) creative domains. Domain scores are summed to form a single index of creative achievement. Creative achievement is assumed to reflect not only creative ability, but also motivation, persistence, opportunity, and resources.

While creative achievement and divergent thinking are typically modestly correlated, our previous investigations suggest that there are reliable differences in how creative achievers and divergent thinkers attend to environmental stimuli and process sensory information. Real-world creative achievers appear to have broad or “leaky” attention, as well as leaky sensory filters, as assessed by the P50 event-related potential (ERP; Zabelina et al., submitted, under revision). Divergent thinking, on the other hand, is linked with the ability to focus and shift attention, supporting attentional flexibility, as well as with highly selective sensory filters, as assessed by the P50 ERP (Zabelina et al., submitted, under revision).

It is not surprising that divergent thinking is associated with focused attention. Divergent thinking tests assess the ability to generate new and appropriate responses to a given problem within a limited time—typically within 2–3 min (e.g., Guilford, 1967; Torrance, 1974; Goff and Torrance, 2002). Responses are scored for fluency (number of responses), and originality/novelty of responses, with the total divergent thinking score reflecting a weighted total of fluency and originality combined, as suggested by the scoring manual (Goff and Torrance, 2002; also see Guilford, 1950; Runco and Acar, 2012). Therefore, people who are able to quickly provide a response, inhibit the just-given response, and quickly move on to the next response are the ones with the higher divergent thinking scores. Indeed, divergent thinking scored by this method has recently been suggested to depend on the overall executive processes (Gilhooly et al., 2007; Nusbaum and Silvia, 2011; De Dreu et al., 2012; Wiley and Jarosz, 2012), i.e., general-purpose control mechanisms such as the ability of the cognitive system to configure itself for the performance of specific task goals (Botvinick et al., 2001; Miyake and Friedman, 2012).

An alternative methods of scoring divergent thinking tests is the Consensual Assessment Technique (CAT; Amabile, 1982). Here independent judges subjectively rate each participant's responses according to their own notion of “creativity.” We employed both the standard scoring method based on the manual, as well as the CAT method to score our divergent thinking tests.

Real-world creative achievements, on the other hand, may reflect a different type of creativity, as they encompass more than just the ability to think in a divergent manner. There are many differences between timed laboratory tests of divergent thinking and real world creative achievement. The latter requires both the generation of an original idea and some level of investment into its further development. Differences between measures of divergent thinking and creative achievement therefore reflect differences in the time course of the process, motivation, resources, and other factors.

In the current study we examine whether sub-clinical levels of psychopathology in a healthy non-clinical sample are associated with real-world creative achievement (CAQ: Carson et al., 2005) or divergent thinking (Goff and Torrance, 2002). Based on our prior results, we expected divergent thinking scored with the manual method and real-world creative achievements to differentially relate to psychopathology-spectrum measures. Divergent thinking scored with the CAT method and creative achievement, on the other hand, should show similar pattern of results, given that the CAT method emphasizes general creativity. First, we examined the internal structure of our psychopathology measures, and, in particular, evaluated the case for a strong general factor(s) (Study 1)—this was done in order to evaluate the perception that creativity is associated with “madness.” We then investigated whether the psychopathology-spectrum measures often found to be associated with creativity differentially predict divergent thinking and creative achievement (Study 2).

An important feature of creative ability is intelligence (Sternberg and O'Hara, 1999), as the literature consistently reports a positive association between intelligence and creativity (Batey and Furnham, 2006; Kim et al., 2010). To account for this association, we used academic achievement test percentile scores (Scholastic Assessment Test (SAT) or American College Testing (ACT); College Board, 2012; ACT Inc., 2014) as a proxy for general intelligence to factor out a general common factor between creative achievement, divergent thinking, and intelligence.

## Study 1

### Methods

#### Participants

One hundred participants ages 18–30 (mean age = 20.55, *SD* = 2.51, male/female = 33/67) took part in the present study. None of the participants had been hospitalized for psychiatric or neurological reasons, and none abused alcohol or drugs. Two participants had history of depression (one in the past, but in remission at the time of the study; one current, treated with Zoloft); one had dysthymia (current, but not taking medication); one had mild anxiety (current, no medication). Seven participants had first-degree relatives with diagnosed psychiatric illnesses. The relations were: a sister with Bipolar I Disorder, anxiety, and psychotic features (auditory hallucinations); a mother with mild depression; a father with depression; a mother with depression; a mother with depression; a twin sister with depression; a mother with Bipolar Disorder, and a father with depression.

All subjects were Caucasian, and right-handed, as assessed by the Chapman Handedness Questionnaire (Chapman and Chapman, 1987). Participants completed an informed consent prior to participating in the study and received $20 for their participation. The study was approved by the Institutional Review Board of Northwestern University.

#### Procedure

Participants were tested individually, with each session lasting up to 2 h, as part of a larger experimental session. Participants first completed the divergent thinking test, followed by the battery of questionnaires. Other tests were administered as part of the study, such as the Compound Remote Associates (CRA) test, but data did not prove to be reliable, and therefore are not included in this report.

#### Measures

*Schizotypal Personality Questionnaire* (SPQ: Raine, 1991) is a self-report scale modeled on DSM-III-R criteria for shizotypal personality disorder. The SPQ consists of twenty-two items with binary choice responses: “yes” and “no.” The SPQ has high sampling validity, high internal and test-retest reliability, convergent, discriminant, and criterion validity (Raine, 1991). Example statements include “I am an odd, unusual person,” and “I feel I have to be on my guard even with friends.” One participant had missing SPQ data. The mean SPQ score was 7.84 (*SD* = 4.84, range 0–19).

*The Psychoticism Scale of the PID-5* (PID5-P: Krueger et al., 2011) was developed for the DSM-5 in order to assess traits that may or may not constitute a formal personality disorder. The PID5-P consists of 34 statements that are answered on a 4-item Likert scale, from “Very often or often false” to “Very true or often true.” Example statements include “I often have thoughts that make sense to me but that other people say are strange,” and “Sometimes I get this weird feeling that parts of my body feel like they're dead or not really me.” The mean PID5-P score was 1.72 (*SD* = 0.46, range 1.0–3.0).

*Hypomanic Personality Scale* (HPS: Eckblad and Chapman, 1986) is designed to identify people with hypomanic personality. The HPS consists of 48 statements with binary choice responses: “True” and “False.” Example statements include “I am frequently in such high spirits that I can't concentrate on any one thing for too long,” and “My moods do not seem to fluctuate any more than most people's do (reverse-scored).” The mean HPS score was 16.18 (*SD* = 7.31, range 3–36).

*Adult ADHD Self-Report Scale* (ASRS-vI.I: Kessler et al., 2005) scale is consistent with DSM-IV criteria and addresses the manifestations of ADHD symptoms in adults. It consists of eighteen questions, and is answered on a 5-item Likert scale, from “Never” to “Very often.” Example questions include “How often do you leave your seat in meetings or other situations in which you are expected to remain seated?” and “How often do you make careless mistakes when you have to work on a boring or difficult project?” The ADHD mean score was 2.23 (*SD* = 0.53, range 1.4–4.3).

*Beck Depression Inventory* (BDI: Beck et al., 1996) is designed to reflect how a person is feeling at the moment, and comprises twenty items, with 4–7 choices per item. Example statements include: “Sadness: I do not feel sad (0), I feel sad much of the time (1), I am sad all the time (2), I am so sad or unhappy that I can't stand it (3),” and “Loss of interest: I have not lost interest in other people or activities (0), I am less interested in other people or things than before (1), I have lost most of my interest in other people or things (2), It's hard to get interested in anything (3).” Four participants had missing BDI data. The BDI mean score was 9.97 (*SD* = 7.39, range 0–30).

*Autism-Spectrum Quotient* (ASQ; Baron-Cohen et al., 2001) assesses the degree to which adults with normal intelligence have traits associated with the autistic spectrum. The ASQ consists of 50 questions, with four response options from “definitely agree” to “slightly disagree.” Approximately half of the statements score 1 point for “definitely agree” or “slightly agree” responses, while the other half of the statements score 1 point for “definitely disagree” or “slightly disagree” responses. The ASQ measure exhibits good test-retest and inter-rater reliability. Example statements include “I prefer to do things the same way over and over again” and “I enjoy social chit-chat (reverse-scored).” One participant had missing ASQ data. The ASQ mean score was 17.85 (*SD* = 6.45, range = 5–35).

### Analysis

Internal consistencies and general factor saturation for each of the psychopathology scales was assessed using the Pearson correlations between items to calculate the α, ω total, and ω hierarchical coefficients (Zinbarg et al., 2005; Revelle and Zinbarg, 2009; Revelle, 2014). Given the absence of *a priori* predictions regarding the underlying structure of these scales, latent variable exploratory factor analyses were conducted based on responses to all the items of the six dimensional psychopathology measures. These EFAs were based on the Pearson correlations between scored responses using Ordinary Least Squares regression models with oblique rotation (Revelle, 2014). Factor solutions were considered for EFAs, which extracted between 1 and 10 factors. Goodness-of-fit was evaluated using the “nfactors” function in the *psych* package (Revelle, 2014) in the R computing environment (R Core Team, 2014), which generates fit statistics based on a wide range of methods, including the Root Mean Squared Error of Approximation (RMSEA; Hu and Bentler, 1999), the empirically-derived root mean square of the residual corrected for degrees of freedom (Kenny, 2014), and the Bayesian Information Criterion (BIC; Kenny, 2014). Evaluation of the factor structure also made use of parallel analyses, which compares “scree” plots of the eigenvalues based on observed data with those from a random matrix of simulated data of the same size and number of observations (Revelle, 2014). It should be noted that 200 or more pairwise administrations between items are recommended when conducting exploratory factor analyses of this nature as smaller samples will often suffer from instability among the correlations. Evaluation of the KMO measure of sampling adequacy (Kaiser and Rice, 1974) demonstrated that the correlation matrix was not invertible, a circumstance which frequently results from instability. As such, the results of the EFAs reported here should be considered preliminary rather than conclusive. In addition, mean item communalities have been included with the fit statistics for each of the factor solutions shown.

### Results

Internal consistencies for each of the psychopathology measures are reported in Table 1. The α values were high for all of the scales, ranging from 0.80 to 0.94, and these values were generally consistent with the ω total values. Values for the ω hierarchical measure of general factor saturation varied considerably, ranging from low values of 0.45 and 0.49 for the ASQ and SPQ, respectively, to relatively high values of 0.66 and 0.68 for the PID5-P and the BDI.

**Table 1:** **Alpha, omega hierarchical and omega total for the psychopathology scales**.

	α	ω **hierarchical**	ω **total**	**Items**
ADHD	0.85	0.56	0.88	18
ASQ	0.86	0.45	0.89	50
BDI	0.85	0.68	0.88	20
HPS	0.80	0.63	0.83	48
PID5-P	0.94	0.66	0.95	33
SPQ	0.83	0.49	0.86	22

**Figure 1:**
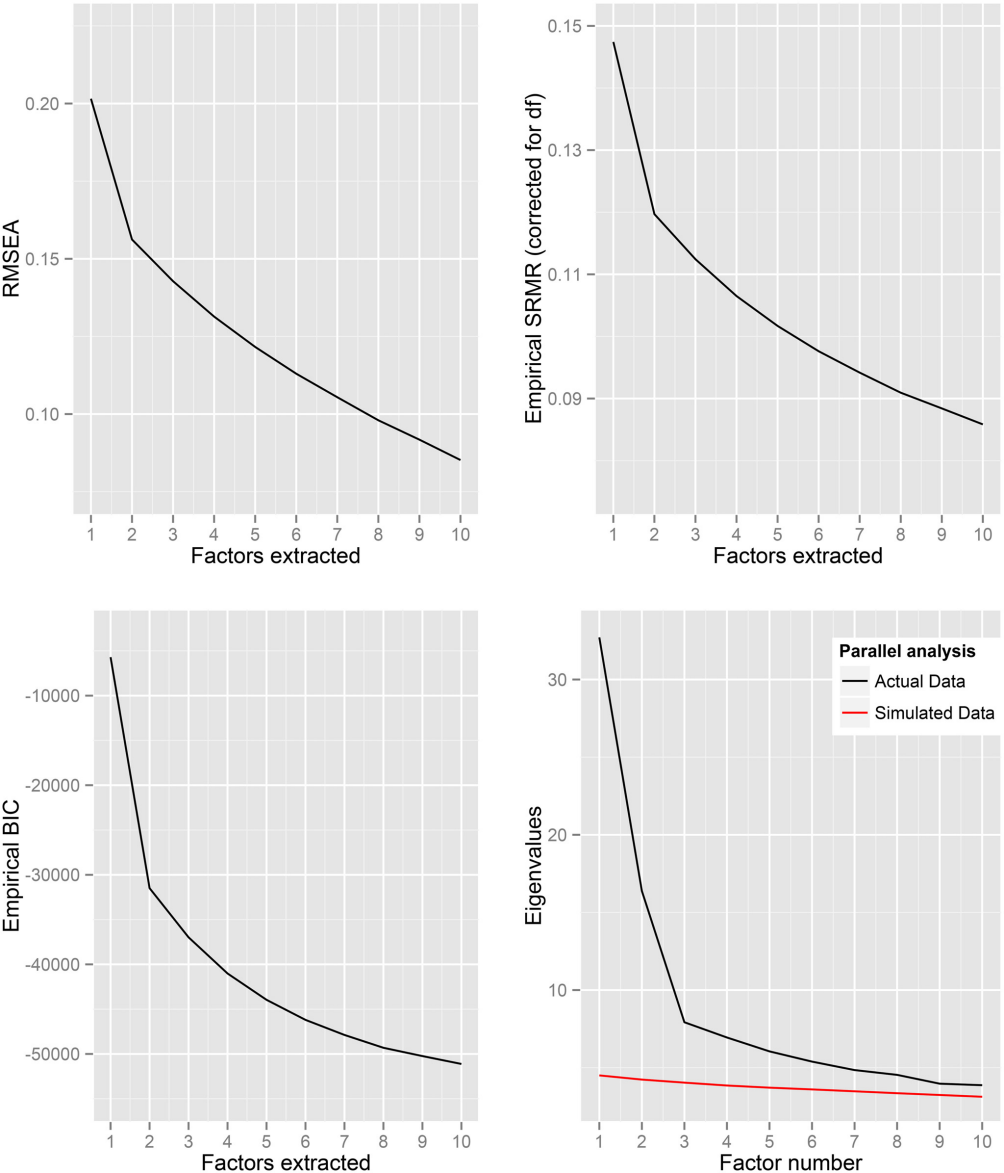
**Fit statistics and eigenvalues based on extraction of 1 to 10 factors from the correlations between all of the psychopathology measures**.

Fit statistics are reported in Table 2 based on the extraction of 1–10 factors from the correlations of scores between items in all six of the psychopathology scales. Figure 1 depicts plots of the fit statistics as well as the eigenvalues for the actual and simulated data. Both the RMSEA and the empirically-derived root mean square residual suggest that none of the factor solutions provide a strong fit. This is consistent with the BIC, which does not reach a localized minimum at fewer than 10 factors, and the parallel analysis, for which the eigenvalues based on factoring of the actual data fail to cross below those which would be expected based on simulated random data.

**Table 2:** **Fit statistics based on extraction of 1 to 10 factors**.

**Factors extracted**	**RMSEA**	**eBIC**	**eSRMR**	**Mean *h*^2^**
1	0.205	−5.708	0.147	0.18
2	0.158	−31.491	0.118	0.27
3	0.143	−36.976	0.111	0.31
4	0.131	−41.013	0.104	0.35
5	0.122	−43.955	0.099	0.39
6	0.113	−46.195	0.095	0.42
7	0.105	−47.877	0.091	0.45
8	0.098	−49.312	0.087	0.48
9	0.092	−50.236	0.084	0.50
10	0.085	−51.115	0.081	0.53

*RMSEA, root mean square error of approximation; eBIC, empirically-derived Bayesian Information Criterion; eSRMR, empirically-derived root mean square of the residual corrected for degrees of freedom. mean h^2^ is the mean communality across items in the solution where communality is the sum of the squared loadings*.

Visual inspection of the plots in Figure 1 provide some evidence to support the two (and perhaps three) factor solution(s). Table 3 shows the most highly loaded items for each factor of the two-factor solution. While the organization and loadings of the items varied according to the number of factors extracted, factors with similar content (“Unusual behavior” and “Social awkwardness”) were found in each of the factor solutions from 3 to 10 factors. The highest loaded items in the third factor for the three factor solution were “I am frequently so ‘hyper’ that my friends kiddingly ask me what drug I'm taking,” “I am considered to be kind of a ‘hyper’ person,” and “People often look at me as if I'd said something really weird.”

**Table 3:** **Most highly loaded items for each factor of the two-factor solution**.

**Item**	**Loading**	**Scale**
**FACTOR 1**		
Other people seem to think my behavior is weird.	0.75	PID5-P
Others seem to think I'm quite odd or unusual.	0.75	PID5-P
People often look at me as if I'd said something really weird.	0.72	PID5-P
I often have ideas that are too unusual to explain to anyone.	0.70	PID5-P
I often have thoughts that make sense to me but that other people say are strange.	0.68	PID5-P
My thoughts are strange and unpredictable.	0.68	PID5-P
People have told me that I think about things in a really strange way.	0.67	PID5-P
Have you had experiences with astrology, seeing the future, UFOs, ESP or a sixth sense?	0.67	Schizotypy
I think about things in odd ways that don't make sense to most people.	0.65	PID5-P
My thoughts often don't make sense to others.	0.65	PID5-P
**FACTOR 2**		
I tend to keep in the background on social occasions.	−0.81	SPQ
I find social situations easy.	0.79	ASQ
When I go to a gathering where I don't know anyone, it usually takes me a while to feel comfortable.	−0.77	HPS
I am good at social chit-chat.	0.75	ASQ
I feel very uncomfortable in social situations involving unfamiliar people.	−0.73	SPQ
I feel very uneasy talking to people I do not know well.	−0.72	SPQ
I enjoy social occasions.	0.72	ASQ
At social gatherings, I am usually the “life of the party.”	0.70	HPS
I find it hard to make new friends.	−0.68	ASQ
New situations make me anxious.	−0.67	ASQ

## Discussion and study 2

While the traditional measure of internal consistency (alpha) was more than adequate for all of the scales independently, the evidence for a strong general factor was low for the ASQ scale, the SPQ scale, and, to a lesser extent, the ADHD scale. This suggests that these scales have multidimensional factor structures by themselves and that the use of single scale level scores for these measures will not distinguish between variability across the underlying constructs. While the presence of low general factor saturation on some measures does not allow for any conclusions to be drawn about the factor structure underlying the full set of items across all the scales, it does support the need for further investigation of structure across and within scales.

Analyses of the factor structure across the scales were largely inconclusive. None of the factor solutions between 1 and 10 factors provided a strong fit with the data based on the most commonly used metrics. For the RMSEA and the empirically-derived root mean square of the residual, only the 10 factor extraction began to approach mediocre fit values (Kenny, 2014). BIC values improved (as expected) as more factors were extracted, but failed to reach a local minimum. This implied that more than 10 factors are needed to fit the items of these six psychopathology scales.

Examination of the item content in the two most consistent factors showed that one of these mapped onto the PID5-Psychoticism scale and the second was comprised of sociability items from a wide variety of scales. Given the poor fit of these factor analytic solutions and the content of the resultant factors, there was little justification for the prospect of correlating creative achievement and divergent thinking scores with factors scores derived from joint administration of these six psychopathology scales. This does not, however, preclude the possibility of evaluating the relationship between the scale scores for these six constructs, creative achievement, and divergent thinking.

### Methods

Study 2 included the same participants, procedure, and methods as in Study 1. In addition, Study 2 incorporated divergent thinking, real-world creative achievement, and academic achievement scores.

#### Measures

***Abbreviated torrance test for adults (ATTA: Goff and Torrance, 2002)***. Divergent thinking was assessed by the Abbreviated Torrance Test for Adults (ATTA: Goff and Torrance, 2002)—a shortened form of the Torrance Test of Creative Thinking (Torrance, 1974). The ATTA consists of three activities (3 min each), one involving written responses (e.g., naming problems that may arise from being able to walk on air or fly without being in an airplane or a similar vehicle), and two involving figural responses (e.g., using incomplete figures to make pictures).

Responses were scored in the standard way of scoring the ATTA according to the manual (Goff and Torrance, 2002). Here, responses were scored for fluency (i.e., a count of the number of pertinent responses), and originality (i.e., the number of responses that are unique or original), with the total scores summed across the three activities (Goff and Torrance, 2002). We computed a total divergent thinking (ATTA manual) score by summing fluency plus two times originality (to equally weight the two scores, since the average fluency score [14.1] was approximately double the average originality score [7.2]. See Runco and Acar (2012) for suggestions on scoring divergent thinking tests). Note that this scoring methods takes into account the number of responses generated by participants, as well as the originality of responses. Two participants had missing ATTA scores.

In addition to scoring ATTA responses according to the manual, responses were also scored with the consensual assessment technique (CAT; Amabile, 1982). Four independent raters (all female) ranked the responses of each participant on the Likert scale (1 = not at all creative, 5 = very creative), from which a total divergent thinking (ATTA CAT) score was derived. The raters were of the same cohort as the participants (19–25 years old), and agreed in their ratings (Cronbach's Alpha = 0.87). Note that the CAT technique's focus is on the subjective creativity of responses, without taking into account the number of responses generated by participants.

***Creative achievement questionnaire (CAQ: Carson et al., 2005)***. We assessed real-world creative behavior with the Creative Achievement Questionnaire, a survey on which participants cataloged any prior creative achievements across ten creative domains (visual art, music, dance, architectural design, creative writing, humor, inventions, scientific discovery, theater and film, and culinary arts). In the Music domain, for example, questions range from “I have no training or recognized talent in this area” (score of 0) to “My compositions have been critiqued in a national publication” (score of 7). In the Scientific Discovery subset, scores vary from “I have no training or recognized ability in this field” (score of 0) to “My work has been cited by other scientists in national publications” (score of 7). Separate domain scores were then combined to form a single index of creative achievement (*M* = 13.66, *SD* = 11.08, min = 0, max = 48). One participant had missing CAQ data. CAQ scores were positively skewed, therefore we used the signed log transformation to normalize the CAQ distribution.

***Academic test scores***. Participants provided their SAT or ACT scores, depending on which achievement test they took. These were converted into percentile scores based on the national statistics in 2012 (*M* = 97.94, *SD* = 2.20, min = 87, max = 100; College Board, 2012; ACT Inc., 2014). In prior studies in our laboratory, self-reported scores were confirmed with actual scores through the admissions office, and the two correlated *r* = 0.97 (Wegbreit et al., 2012). Twenty-eight people did not report their academic test scores (therefore degrees of freedom will be different when academic test scores are included in the analyses).

### Analysis

The goal of Study 2 was to determine whether psychopathology-spectrum scales predict, independently, creative achievement and divergent thinking. Given that there was no clear underlying common structure within the psychopathology-spectrum scales, we performed separate linear regression analyses predicting divergent thinking and creative achievement, while controlling for academic achievement scores.

Given prior findings in the literature, as well as our previous investigations suggesting that creative achievement is associated with leaky attention, as well as with reduced sensory gating, we expected traits associated with psychosis, such as schizotypy (SPQ) and psychoticism (PID5-P), to predict creative achievement. We also reasoned that hypomania (HPS) should predict creative achievement, given prior evidence (Furnham et al., 2008), and that drive and energy are needed to have a large number of creative achievements in the real world (especially in our undergraduate sample).

Our previous investigations also suggest that divergent thinking is associated with selective attention, as well as with more selective sensory gating, therefore we did not expect divergent thinking to relate to any psychopathology-spectrum measures.

### Results

#### Psychopathology spectrum traits and creativity

Zero-order correlations between psychopathology-spectrum scales, creative achievement, divergent thinking (ATTA manual and ATTA CAT), and academic achievement scores are reported in Table 4, along with the 95% confidence intervals of the correlations. The correlation between creative achievement and divergent thinking scored manually did not significantly differ from zero, though the correlation was significant between creative achievement and divergent thinking when scored with the consensual assessment technique (*r* = 0.32, *p* < 0.01). Both scoring methods were significantly associated with academic achievement scores (ATTA CAT *r* = 0.22, *p* = 0.01; ATTA manual *r* = 0.19, *p* = 0.03). There was no association between creative achievement and academic achievement scores.

**Table 4:** **Correlations among academic test scores (Ach Tests), divergent thinking (ATTA), and creative achievement (CAQ)**.

	**Achievement tests**	**ATTA manual**	**ATTA CAT**	**CAQ**	**PID5P**	**SPQ**	**ADHD**	**BDI**	**HPS**
ATTA Man.	0.19								
	(0.03–0.36)								
ATTA CAT	0.22	0.56							
	(0.04–0.37)	(0.43–0.68)							
CAQ	0.02	0.15	0.32						
	(−0.14–0.20)	(−0.04–0.36)	(0.15–0.47)						
PID5P	−0.04	0.19	0.24	0.29					
	(−0.23–0.15)	(0.00–0.38)	(0.05–0.42)	(0.13–0.46)					
SPQ	−0.01	0.06	0.09	0.15	0.72				
	(−0.21–0.26)	(−0.13–0.24)	(−0.09–0.30)	(−0.03–0.34)	(0.63–0.79)				
ADHD	0.07	0.02	0.00	0.25	0.61	0.41			
	(−0.09–0.25)	(−0.18–0.23)	(−0.20–0.18)	(0.06–0.44)	(0.48–0.70)	(0.25–0.52)			
BDI	−0.11	−0.10	−0.03	0.06	0.36	0.39	0.38		
	(−0.31–0.06)	(−0.28–0.12)	(−0.21–0.20)	(−0.12–0.23)	(0.22–0.54)	(0.21–0.58)	(0.27–0.54)		
HPS	0.07	0.26	0.34	0.43	0.51	0.19	0.42	0.12	
	(−0.07–0.21)	(0.07–0.47)	(0.19–0.49)	(0.27–0.56)	(0.31–0.67)	(0.02–0.38)	(0.21–0.59)	(−0.08–0.28)	
ASQ	0.15	0.17	0.18	0.06	−0.38	−0.61	−0.26	−0.40	0.11
	(−0.12–0.36)	(−0.02–0.35)	(−0.02–0.41)	(–0.13–0.23)	(−0.52– −0.23)	(−0.72– −0.47)	(−0.41– −0.10)	(−0.56– −0.23)	(−0.06–0.27)

*Values in parentheses indicate the 95% confidence interval of the correlations*.

With respect to the psychopathology spectrum scales, creative achievement was significantly correlated with HPS (*r* = 0.43, *p* < 0.001), PID5-P (*r* = 0.29, *p* < 0.01), and ADHD (*r* = 0.25, *p* = 0.01). Both methods of scoring divergent thinking were significantly correlated with HPS (ATTA manual *r* = 0.26, *p* = 0.02; ATTA CAT *r* = 0.34, *p* < 0.001). Only the consensual assessment technique for scoring divergent thinking was significantly correlated with the PID5P (*r* = 0.24, *p* = 0.03).

#### Multiple regression analyses controlling for academic achievement scores

Given that there was no clear underlying common structure between the psychopathology-spectrum scales, we performed separate linear regression analyses predicting creative achievement and divergent thinking, while controlling for academic achievement scores.

As expected, creative achievement was significantly predicted (after controlling for achievement test scores) by the PID5-P, *t*_(83)_ = 2.69, *p* = 0.01, *b* = 0.28; and the HPS, *t*_(83)_ = 4.16, *p* < 0.001, *b* = 0.44 (Table 5).

**Table 5:** **Creative achievement as a function of psychopathology-spectrum scales, controlling for academic achievement scores**.

**Variable**	***b***	**SE *b***	***t***	***p***
SPQ	0.15	0.11	1.42	0.16
SAT/ACT	0.03	0.11	0.25	0.80
PID5P	0.28	0.1	2.69	0.01^**^
SAT/ACT	0.03	0.1	0.31	0.76
HPS	0.44	0.1	4.16	0.00^**^
SAT/ACT	0	0.1	0.01	0.99
ADHD	0.21	0.11	1.92	0.06
SAT/ACT	0	0.11	0.02	0.98
BDI	0.01	0.12	0.05	0.96
SAT/ACT	0.02	0.11	0.18	0.86
ASQ	0.06	0.11	0.56	0.58
SAT/ACT	0.01	0.11	0.11	0.92

^*^<0.05,

** *<0.01*.

Controlling for achievement test scores, divergent thinking when scored with the consensual assessment technique was also significantly predicted by the PID5-P, *t*_(82)_ = 2.44, *p* = 0.02, *b* = 0.25, and the HPS, *t*_(82)_ = 3.16, *p* < 0.001, *b* = 0.33 (Table 6). When scored with the traditional manual method, divergent thinking was not significantly predicted by any of the psychopathology measures (Table 7).

**Table 6:** **Divergent thinking scored with the consensual assessment technique as a function of psychopathology-spectrum scales, controlling for academic achievement scores**.

**Variable**	***b***	**SE *b***	***t***	***p***
SPQ	0.09	0.11	0.78	0.44
SAT/ACT	0.22	0.11	2.03	0.05^*^
PID5P	0.25	0.1	2.44	0.02^*^
SAT/ACT	0.24	0.11	2.2	0.03^*^
HPS	0.33	0.1	3.16	0.00^**^
SAT/ACT	0.2	0.1	1.92	0.06
ADHD	−0.01	0.11	−0.13	0.90
SAT/ACT	0.22	0.11	2.04	0.04^*^
BDI	0.06	0.12	0.55	0.59
SAT/ACT	0.24	0.11	2.17	0.03^*^
ASQ	0.14	0.11	1.33	0.19
SAT/ACT	0.2	0.11	1.84	0.07

* *<0.05*,

** *<0.01*.

**Table 7:** **Divergent thinking scored with the manual scoring method as a function of psychopathology-spectrum scales, controlling for academic achievement scores**.

**Variable**	***b***	**SE *b***	***t***	***p***
SPQ	0.05	0.11	0.42	0.67
SAT/ACT	0.2	0.11	1.85	0.07
PID5P	0.16	0.1	1.52	0.13
SAT/ACT	0.2	0.11	1.87	0.07
HPS	0.19	0.1	1.8	0.08
SAT/ACT	0.18	0.11	1.68	0.10
ADHD	0.1	0.11	0.11	0.91
SAT/ACT	0.19	0.11	1.77	0.08
BDI	0.01	0.11	0.11	0.91
SAT/ACT	0.21	0.11	1.94	0.06
ASQ	0.14	0.11	1.3	0.20
SAT/ACT	0.17	0.11	1.59	0.12

^*^<0.05, ^**^<0.01.

### Discussion

Here we systematically examined the presence of an underlying common structure within the psychopathology-spectrum scales often postulated to be associated with creativity (Study 1), and investigated whether these scales are associated with two aspects of creativity: 1. real-world creative achievement, and divergent thinking, a laboratory measure of creative cognition, scored by two different methods (Study 2).

Latent variable exploratory factor analyses of the factor structure across the scales were largely inconclusive. Examination of the item content in the two most consistent factors showed that one of these mapped onto the PID5-Psychoticism scale and the second was comprised of sociability items from a wide variety of scales. Given the poor fit of these factor analytic solutions, there was little justification for the prospect of correlating creative achievement and divergent thinking scores with factor scores. We therefore evaluated the relationship between the psychopathology-spectrum scale scores, creative achievement, and divergent thinking within separate multiple regression analyses. Controlling for academic achievement, real-world creative achievement was significantly predicted by psychoticism and hypomania. The association between real-world creative achievement and psychoticism supports the suggestion that milder forms of psychopathology, such as sub-clinical levels of psychoticism may indeed be adaptive for creativity (O'Reilly et al., 2001), while clinical levels of these disorders, such as psychosis, would presumably be maladaptive.

Creative achievement was predicted by psychochoticism, however, it did not relate to schizotypy in our sample, as it has in prior studies (Kinney et al., 2001; Abraham and Windmann, 2008). This result indicates that traits associated with psychoticism, such as impulsivity and sensation-seeking, may benefit creative achievement.

Creative achievement was also predicted by hypomania, indicating that high energy levels are associated with increased creative achievement in the real world. To be clear, predisposition to mental illness is neither *necessary* nor *sufficient* for creative achievement. There are numerous eminent creative people without mental illness, and multiple possibilities can explain the relationship between mental illness and creative eminence.

Divergent thinking scored with the CAT scoring method, which taps into the overall creativity of participants' responses, without taking into account the number of responses produced by participants, was reliably predicted by psychoticism and hypomania, controlling for academic achievement scores. Additionally, the CAT divergent thinking and creative achievement significantly correlated, whereas divergent thinking scored with the manual and creative achievement showed no reliable association. These results indicate that divergent thinking scored with the CAT technique is more closely linked with creativity in the real world.

Divergent thinking scored with the manual scoring method was not reliably predicted by any of the psychopathology-spectrum scales, and only marginally predicted by hypomania and autism-spectrum. Given that the manual scoring emphasizes not only the originality of participants' responses, but also their total number within a limited time, divergent thinking scored with this method may tap into the executive processes that are needed to perform well on timed laboratory tests, where performance may be impeded by having sub-clinical forms of psychopathology.

Indeed, although undeniably a feature of the creative process, producing numerous responses on a divergent thinking test appears to be more executive in nature than previously thought (Nusbaum and Silvia, 2011). Divergent thinking, for example, is found to rely on focused attention (Zabelina et al., under revision), and selective sensory filters (Zabelina et al., submitted). Additionally, executive functions “updating,” which is closely associated with the concept of working memory (Jonides and Smith, 1997), and “inhibition,” or the ability to suppress a dominant, but irrelevant response (Miyake and Friedman, 2012), significantly predict divergent thinking, while “shifting”—the process of switching between different tasks or mental sets (Monsell, 1996), does not (Benedek et al., 2014). Divergent thinking is also found to correlate with inhibition defined either by performance on the Stroop task (Groborz and Neçka, 2003; Edl et al., 2014), or the random motor generation task (Benedek et al., 2012; Zabelina et al., 2012). Thus, it is not surprising that we did not find an association between the divergent thinking test (where the score is comprised of fluency and originality of responses) and sub-clinical levels of psychopathology.

Although it has been suggested that depressive states may be conducive to creativity by narrowing the focus of attention and selecting the most practical ideas to pursue, or persistence in confronting problems (Verhaeghen et al., 2005), we found that neither divergent thinking nor creative achievement in our sample was associated with depression.

There were several limitations to this study. First, findings from the analyses are limited by the sample size. Second, it is important to recognize that there are other features of psychopathology that may relate to creativity, such as personality trait Openness to Experience (Miller and Tal, 2007; DeYoung et al., 2012). Future studies will need to investigate the relationship between Openness and other “normal-range” personality traits with both creative achievement and psychopathology. Finally, both psychopathology and creative achievement would ideally be measured by informants. Future research should make use of such measures, although historically such measures have not been widely available.

## Conclusion

Here we examined the associations between psychopathology-spectrum measures and creativity. The factor structure of psychopathology measures revealed no common underlying factors, based on the most commonly used metrics. Separate linear regression analyses revealed that, after controlling for academic achievement, psychoticism and hypomania reliably predicted real-world creativity, as well as subjective ratings of creativity on the divergent thinking test. None of the psychopathology-spectrum scales reliably predicted scores on the timed divergent thinking scored with the manual method. The link between creativity and psychopathology requires additional investigation to more precisely reveal the cognitive mechanisms that both unite and distinguish creative people from those with a psychiatric disorder.

### Conflict of interest statement

The authors declare that the research was conducted in the absence of any commercial or financial relationships that could be construed as a potential conflict of interest.
